# PREFACE

**DOI:** 10.2174/1570159X1201140117155800

**Published:** 2014-01

**Authors:** Tom Salt

*Current Neuropharmacology* enters its 12th volume after having increased the number of issues to six per year. This reflects the increasing number of quality papers that are received for publication. In addition to providing the opportunity for more papers to be published, it also allows for a more rapid publication schedule. All of these are features of a thriving journal, but a thriving journal would not be possible without the dedicated efforts throughout the year of authors, Editorial Board members, Special Guest Editors, anonymous reviewers, and of course the efforts of Bentham Science Publishers. My thanks go out to all of you. 

The diversity and high standard of work published in *Current Neuropharmacology* is reflected in the content of the present issue of the journal which contains a wide range of topical reviews. Subjects covered are the *Therapeutic Strategies for Alzheimer’s Disease* (Agis-Torres *et al.*); *Purinergic Signaling and Long-Term Potentiation* (DÜster *et al.*); *Pregabalin in Neuropathic Pain* (Verma *et al.*); Ketamine as an Antidepressant (Hasselmann); *Ionotropic Glutamate Receptors in Childhood Neurodevelopmental Disorders* (Uzunova *et al.*). The journal continues to offer an excellent vehicle for the publication of novel reviews and hypotheses. 

